# Posttraumatic stress disorder symptoms among healthcare workers during the Omicron era

**DOI:** 10.3389/fpsyt.2023.1140511

**Published:** 2023-05-24

**Authors:** YuanYuan Yin, Sizhu Han, Jiaoqiong Guan, DuanWei Wang, HaiRong Wang, Ti-Fei Yuan, Ying Yang

**Affiliations:** ^1^Shandong Mental Health Center, Jinan, Shandong, China; ^2^Wenzhou Medical University, School of Mental Health, Wenzhou, Zhejiang, China; ^3^Shanghai Key Laboratory of Psychotic Disorders, Brain Health Institute, National Center for Mental Disorders, Shanghai Mental Health Center, Shanghai Jiao Tong University School of Medicine, Shanghai, China; ^4^Department of Rehabilitation Medicine, Guangzhou First People’s Hospital, School of Medicine, South China University of Technology, Guangzhou, Guangdong, China; ^5^Co-innovation Center of Neuroregeneration, Nantong University, Nantong, China

**Keywords:** exposure to COVID-19, euthymia, posttraumatic stress disorder symptoms, perceived social support, healthcare workers

## Abstract

**Background:**

The COVID-19 pandemic has caused significant psychological stress among healthcare workers. This study aimed to clarify the factors that influenced health workers’ posttraumatic stress disorder (PTSD) symptoms.

**Method:**

A total of 443 healthcare workers from eight Mental Health Centers in Shandong were recruited to attend an online survey. Participants completed self-evaluation measures of exposure to the COVID-19 environment and PTSD symptoms, as well as measures of potential protective factors such as euthymia and perceived social support.

**Results:**

About 45.37% of healthcare workers had severe symptoms of PTSD symptoms. Healthcare workers with more serious PTSD symptoms were significantly related to higher exposure to COVID-19 (*r* = 0.177, *p* < 0.001), as well as lower levels of euthymia (*r* = −0.287, *p* < 0.001) and perceived social support (*r* = −0.236, *p* < 0.001). The structural equation model (SEM) further revealed that the impact of exposure to COVID-19 on PTSD symptoms was partially mediated by euthymia, and moderated by perceived social support, especially from others (e.g., friends, leaders, relatives and colleagues).

**Conclusion:**

These findings suggested that improving the state of euthymia, getting social support from others could alleviate PTSD symptoms among healthcare workers during the COVID-19.

## Introduction

At the beginning of 2022, the Omicron variant rapidly spread in China. As of March 2022, there were 5,559 new cases of asymptomatic infections nationwide. Of these, over 5,000 cases of native infection have been reported, with Shandong Province ranking eighth in terms of the number of increases (1). Shandong, the second most populous province in the country (2), provided a conductive environment for its spread. The outbreak firstly started in Qingdao and quickly spread to other cities in Shandong. Many places implemented strict closure policies to try to control the spread of the virus. By the end of March 2022, the cumulative number of asymptomatic infectors in Shandong had reached 2,175 cases (3).

The COVID-19 pandemic has caused serious mental health problems among the general public (4). Healthcare workers, as a major force in the fight against the COVID-19 pandemic, have been suffering from high risks of infection and increasingly heavy workloads (5, 6). These factors have inevitably caused damage to their mental health. For instance, previous studies have found that healthcare workers had severe anxiety, depression and stress (7, 8). Moreover, a global review study on the mental health of healthcare workers showed that the prevalence of post-traumatic stress disorder (PTSD) in the healthcare population has reached 49%, which was much higher than anxiety (40%) and depression (37%) (9). It was clearly that PTSD symptoms have become a key issue in the mental health of healthcare workers during the COVID-19 pandemic (10).

PTSD could occur after individual experienced a life-threatening trauma. It is a trauma- and stressor-related disorder, the symptoms of which are mainly persistent intrusive memories, avoidance of trauma-related stimuli and hyperarousal to stimuli associated with the traumatic event (11). PTSD could increase the risk of poor physical health (12) and suicide (13, 14). Besides, it has been found to be associated with severe psychiatric co-morbidity (15, 16) and has a long-term negative impact on family life (17). One recent study tracking the mental health of healthcare workers in Guangzhou found that 1 year after the outbreak of COVID-19, healthcare workers still showed symptoms of PTSD, with the prevalence of the disorder even increasing from 10.73 to 20.84% (18). Further, severe PTSD symptoms have been linked to high turnover intention among nurses (19). This could pose a great threat to the health system’s ability to provide adequate care. As a result, it was particularly important to explore the factors that influenced the development of PTSD symptoms.

Overexposure to the COVID-19 pandemic has been proven to be a risk factor for mental health problems (20). Studies have shown that the duration and frequency of exposing to COVID-19-related information were positively associated with levels of anxiety and depression (21). Specifically, overexposure to the COVID-19 information increased individual emotional distress, such as threat, anxiety and depression, and risk perception mediated the relationship between the two factors (20). Some studies have also shown that the negative effect of overexposure to COVID-19 on PTSD symptoms (5, 19, 22), highlighting the mediating role of perceived threats and feelings of vulnerability (22). However, researches on the relationship between exposure to COVID-19 and PTSD symptoms were still lacking. Few studies explored the role of positive psychological states in the process, such as the euthymia and social support. Euthymia is a comprehensive measure of positive mental health (23), which is characterized by the absence of emotional impairment in an individual mental health, flexibility, and resistance to stress (24). Previous researches showed that the Euthymia Scale could detect individual susceptibility to depression (25). The worse the euthymia, the more likely the individual was to develop depressive symptoms. Besides, social support has been proposed to be effective in dealing with mental health impairments (26, 27). For instance, social support could buffer the impact of traumatic events by moderating an individual’s ability to perceive the traumatic event and then to reduce negative thoughts or by increasing an individual’s resources to combat stress (28, 29). As with social support, euthymia may also buffer PTSD symptoms during overexposure to the COVID-19 environment. To this end, the impact of euthymia and social support on the release of PTSD symptoms caused by overexposure to COVID -19 remained to be explored.

To fill in these gaps, we conducted an online survey on healthcare workers to clarify the relationship among exposure to COVID-19, social support, euthymia state and PTSD symptoms. We aimed to provide evidences supporting that social support and euthymia could alleviate the effect of overexposure on PTSD symptoms. This study could provide useful suggestions for the daily care and treatment of healthcare workers, even after the pandemic.

## Methods

### Participants

This online study was conducted among healthcare workers in eight mental health centers in Shandong Province, most of whom were from the psychiatric departments. We chose these centers because the healthcare workers who worked there have had closely contact with patients confirmed to be infected with COVID-19. We contacted around 800 people and eventually received 725 completed online questionnaires. The attrition rate of our study was approximately 9.38%. All of them voluntarily completed the questionnaires and were not paid for their participation. To improve the reliability of results from the subsequent analysis, participants who failed to pass the quality control questions were excluded, leading to a valid rate of 61.10% (443 participants). The study was approved by the Ethics Committee of the Shandong Mental Health Centre. All participants provided informed consent prior to the survey.

#### Questionnaires

To measure healthcare workers’ exposure to COVID-19, we developed a questionnaire including the following questions:(1) Have you ever been informed of a positive test result of COVID-19; (2) Has anyone close to you (e.g., relatives, colleagues, neighbors) ever been confirmed as COVID-19 positive; (3) Have you ever been isolated because you had symptoms of COVID-19 or closely contacted with infected people; (4) Has anyone close to you (e.g., relatives, colleagues, neighbors) ever been isolated because of symptoms of COVID-19 or close contacts; (5) Have you ever worked in a mobile cabin hospital, community or isolated site; (6) Overall, did you think healthcare workers are at higher risk of infection; (7) Which type of controlled area you currently live in; (8) How much time you spent on receiving the information related to COVID-19 every day. The 1–6 questions were scored dichotomously as 1 (yes) or 0 (no). In the seventh question, choosing “Precautionary Zone” was scored as 0, “Controlled Zone” as 1 and “Locked-down Zone” as 2. In the final question, 0 marked for “<30 min/day,” 1 marked for “30–60 min/day,” 2 marked for “60–180 min/day” and 3 marked for “>180 min/day.” This questionnaire has not been subjected to any reliability assessment.

The Impact of Events Scale (IES-R) with 22 items in total was used to assessed the subjects’ PTSD symptoms in the last 7 days after experiencing a traumatic event (30). Here, we indicated the experience relating to COVID-19 as the sole event to be considered. The questionnaire consisted of three different dimensions: avoidance (8 items), intrusion (8 items), and hyperarousal (6 items). Each item had a score of 0 (Not at all) to 4 (Always), with a total score of 0 to 88. The higher the score, the more serious were PTSD symptoms. Among them, those with a total score of greater than 22 were considered to have significant PTSD symptoms (31). In our study, the coefficient of internal consistency (Cronbach’s α) was 0.970.

The Chinese version of the Perceived Social Support Scale (PSSS) was adapted by Jiang Qianjin and his colleagues in 1996 (32), which measured individual perceived social support from two dimensions: family and others (including friends, leaders, relatives and colleagues). There were 12 questions in total and measured on a seven-point Likert scale (1–7, with labels of ‘extremely disagree’ to ‘extremely agree’), with a total score of 12 to 84 (32). The higher total score, the higher level of perceived social support. In the current study, the Cronbach’s *α* was 0.964.

The Euthymia Scale was used to assess individual state of euthymia, contributing to predict positive dimensions of mental health (24). It was multidimensional measurement of psychological well-being and resilience (33). Recently, this scale has been translated into Chinese by Professors Yonggui Yuan and Yuqun Zhang (25). The scale consisted of 10 items, with a score of 1 for “true” and 0 for “false.” The higher the score, the better the individual psychological state. The Cronbach’s α was 0.857 in this study.

### Statistical analysis

The IBM SPSS 26.0 and Mplus 8.3 software were used to analyze data. We divided subjects into low (without obvious PTSD symptoms) or high (with significant PTSD symptoms) groups based on whether the total IES-R score exceeded 22 points. Firstly, we conducted a descriptive statistical analysis of the demographic characteristics between two groups. Secondly, we investigated the relationship between any two of the four factors (i.e., euthymia, perceived social support, exposure to COVID-19 and PTSD symptoms) using Spearman’s rank correlation for all qualified participants. Based on the same data, we constructed a structural equation model to explore the role of euthymia and perceived social support in the relationship between overexposure to COVID-19 and PTSD symptoms. Goodness of the model fit was assessed by comparative fit index (CFI > 0.90), Tucker-Lewis index (TLI > 0.90), root mean square error of approximation (RMSEA<0.08) and standardized root mean residual (SRMR<0.08) (34, 35). The significant threshold was set to *p <* 0.05.

## Results

### Demographic information

As Table 1 showed, the mean age of the 443 participants was 35 (SD = 9.421). Of these, 300 were female (67.72%) and 143 were male (32.28%). 286 participants (64.56%) obtained a bachelor’s degree or higher. 237 participants (53.50%) had an annual income of at least 100,000 RMB. Of these 443 participants, 102 (23.02%) went to the front line (quarantine sites, square cabin hospitals, etc.) to provide support.

**Table 1:** Demographic information and questionnaire measurements.

	Low group(*N* = 242)	High group(*N* = 201)	Statistics	*p*
Gender (%)			*Χ*^2^ = 0.781	0.377
Male	83(34.29%)	61(30.34%)		
Female	159(65.70%)	140(69.65%)		
Education (%)			*Χ*^2^ = 0.002	0.963
Below bachelor’s degree	86(35.53%)	71(35.32%)		
Bachelor or above	156(64.87%)	130(64.67%)		
Income (%)			*Χ*^2^ = 3.327	0.068
<¥100,000/year	103(42.56%)	103(51.24%)		
>¥100,000/year	139(57.43%)	98(48.75%)		
Age (Mean ± SD)	34.36 ± 9.481	36.21 ± 9.269	*t* = −2.061	0.040
IES-R score (Mean ± SD)	8.86 ± 7.367	37.12 ± 13.153	*F* = 799.648	
Intrusion	3.88 ± 3.116	14.36 ± 5.210	*F* = 672.812	<0.001
Avoidance	3.08 ± 3.195	13.45 ± 5.187	*F* = 652.431	<0.001
Hyperarousal	1.88 ± 2.241	9.29 ± 4.210	*F* = 548.506	<0.001
PSSS (Mean ± SD)	67.12 ± 13.039	62.43 ± 11.974	F = 16.267	<0.001
Family	22.74 ± 4.741	21.04 ± 4.582	*F* = 15.972	<0.001
Others	44.37 ± 8.648	41.38 ± 8.059	*F* = 14.589	<0.001
Euthymia (Mean ± SD)	8.67 ± 2.213	7.56 ± 2.700	*F* = 24.179	<0.001

### Groups with high vs. low PTSD symptoms

**Figure 1:**
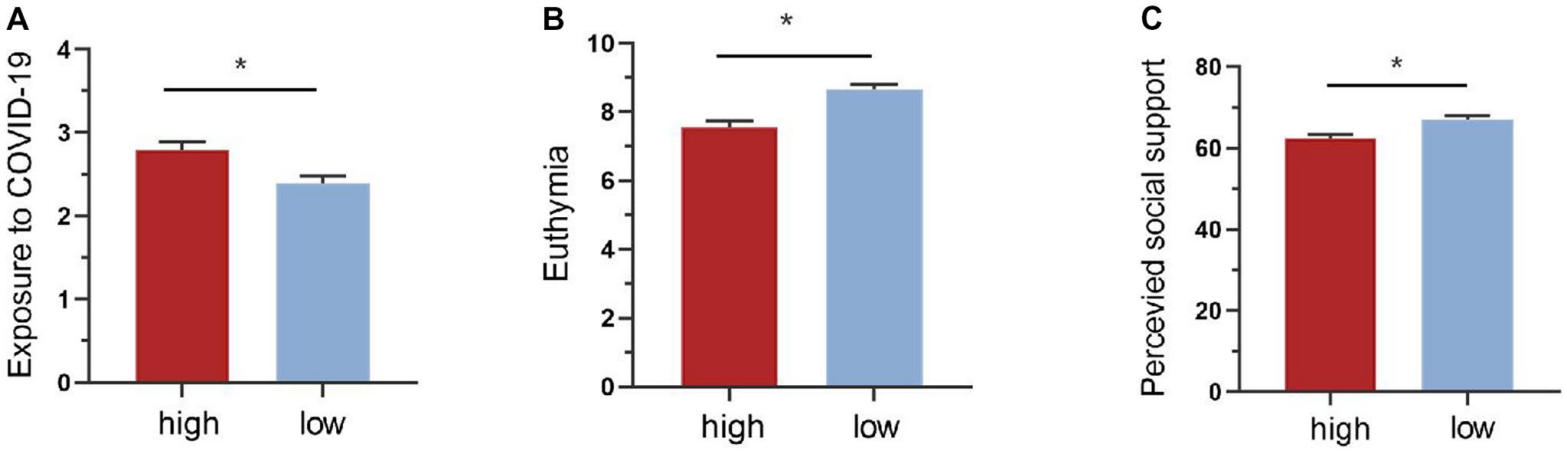
Comparison of groups with low and high PTSD symptoms on exposure, euthymia and perceived social support. **(A)** Comparison of the level of exposure to COVID-19 between groups with low and high PTSD symptoms. The group with high PTSD symptoms showed much more exposure. **(B)** Comparison of euthymia between groups with low and high PTSD symptoms. Compared to the low group, high group decreased significantly on the level of euthymia. **(C)** Comparison of perceived social support between groups with low and high PTSD symptoms. The high group perceived lower level of social support than the low group. *p < 0.05.

According to our criteria, 43.37% of our participants had severe PTSD symptoms, mostly manifesting as intrusion symptoms (Table 1). We then compared demographic statistics between two groups with high or low PTSD symptoms (Table 1). No significant difference was observed on educational year (*Χ*^2^ = 0.002, *p* = 0.963), gender ratio (*Χ*^2^ = 0.781, *p* = 0.377) and annual income (*Χ*^2^ = 3.327, *p* = 0.068). Nevertheless, the mean age of the high group was greater than the low group (*t* = 2.061, *p* = 0.040). To avoid the interpretation of our follow-up results by it, age was controlled as a covariate in the subsequent analysis. We additionally found that greater exposure to COVID-19 (*F* = 8.811, *p* = 0.003), lower euthymia (*F* = 24.179, *p* < 0.001) and perceived social support (*F* = 16.267, *p* < 0.001) in the high group compared to the low group (Figure 1).

### Correlations between PTSD symptoms and other factors

When it comes to correlations among PTSD symptoms and other factors, there was a significant positive correlation between the COVID-19 exposure and PTSD symptoms (*r* = 0.177, *p* < 0.001). Besides, the euthymia (*r* = −0.287, *p* < 0.001) and perceived social support (*r* = −0.236, *p* < 0.001) were also significantly correlated with the level of PTSD symptoms. However, no significant relationship was found between the exposure and perceived social support (*r* = −0.048, *p* = 0.311).

To further explore which aspects of PTSD symptoms were associated with these factors, we then assessed their correlations with each subscale of IES-R. We found higher levels of COVID-19 exposure predicted higher levels of intrusion (*r* = 0.200, *p* < 0.001), avoidance (*r* = 0.138, *p* = 0.004) and hyperarousal (*r* = 0.159, *p* = 0.001). The level of intrusion (*r* = −0.283, *p* < 0.001), avoidance (*r* = −0.250, *p* < 0.001) and hyperarousal (*r* = −0.301, *p* < 0.001) increased when euthymia decreased. Besides, the level of intrusion (*r* = −0.239, *p* < 0.01), avoidance (*r* = −0.204, *p* < 0.001) and hyperarousal (*r* = −0.253, *p* < 0.001) showed significant negative correlations with perceived social support (Table 2).

**Table 2:** Descriptive analysis and inter-correlations of all need variables.

Factors	Mean	SD	EC	Eu	SS	IES	IS	AD	HA
EC	2.58	1.372	1						
Eu	8.16	2.504	−0.143*	1					
PSS	64.99	12.769	−0.048	0.261*	1				
IES	21.68	17.500	0.177*	−0.287*	−0.236*	1			
IS	8.64	6.698	0.200*	−0.283*	−0.239*	0.963*	1		
AD	7.79	6.668	0.138*	−0.250*	−0.204*	0.953*	0.871*	1	
HA	5.25	4.940	0.159*	−0.301*	−0.253*	0.932*	0.872*	0.841*	1

EC, exposure to COVID-19; Eu, euthymia; PSS, perceived social support; IES, impact of events scale; IS, intrusion; AD, avoidance; HA, hyperarousal.

SD, standard deviation. The significant threshold was set to *p* < 0.05. *p < 0.05.

### Structural equation modeling

**Figure 2:**
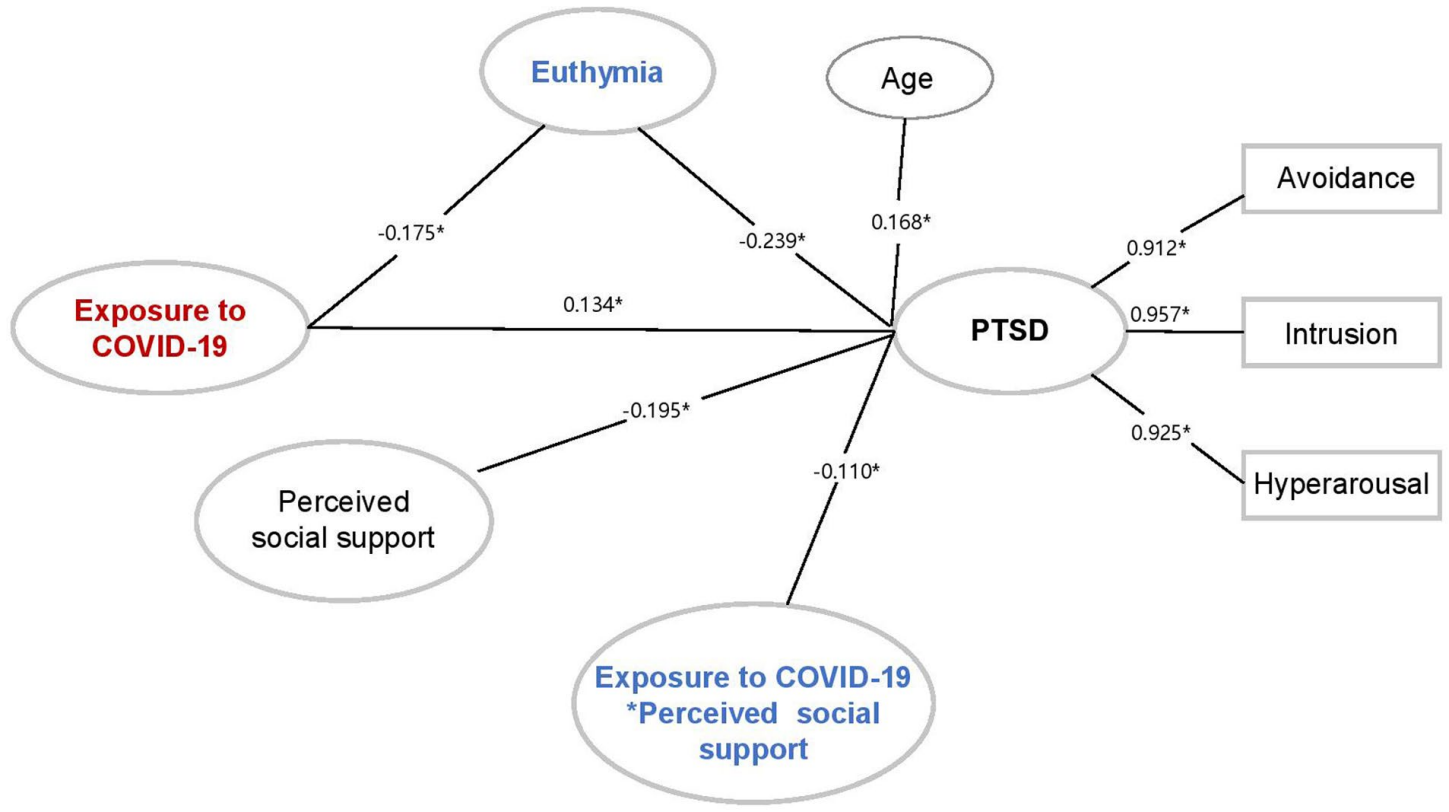
The structural equation model depicting the relationship among exposure to COVID-19, euthymia, perceived social support, and PTSD symptoms. *p < 0.05.

To explore the relationship among the exposure to COVID-19, PTSD symptoms and possible protective effects of euthymia and perceived social support, we developed structural equation models. First of all, we examined the effect of COVID-19 exposure on PTSD symptoms (*β* = 0.813, *p* < 0.001) based on all qualified participants. The IES-R scores served as the potential dependent variable consisting of three dimensions (i.e., avoidance, intrusion and hyperarousal). Then, we investigated the roles of protective factors in this pathway. When euthymia was added to the model, results revealed that euthymia significantly mediated the effect of exposure on PTSD symptoms (*β* = 0.221, *p* = 0.001). That is, the higher exposure to COVID-19 was directly and negatively related to individual euthymia (*β* = −0.319, *p* < 0.001), which was further correlated with more pronounced PTSD symptoms (*β* = −0.692, *p* < 0.001). Overall, the model fitted well with CFI = 0.993, TLI = 0.987, RMSEA =0.056, SRMR = 0.020. After adding social support to the model, the fit remained well with CFI = 0.982, TLI = 0.975, RMSEA =0.062, SRMR = 0.042. Perceived social support was found to moderate the effect of COVID-19 exposure on PTSD symptoms (*β* = −0.110, *p* = 0.030). In particular, healthcare workers who overexposed to COVID-19 tended to show more PTSD symptoms when perceived social support levels were low (*β* = 1.445, *p* = 0.002). However, such effect was decreased at high levels of perceived social support (*β* = 0.171, *p* = 0.643) (Figure 2). Overall, the direct effect of overexposure to COVID-19 on PTSD symptoms decreased when individuals perceived high level of social support.

**Figure 3:**
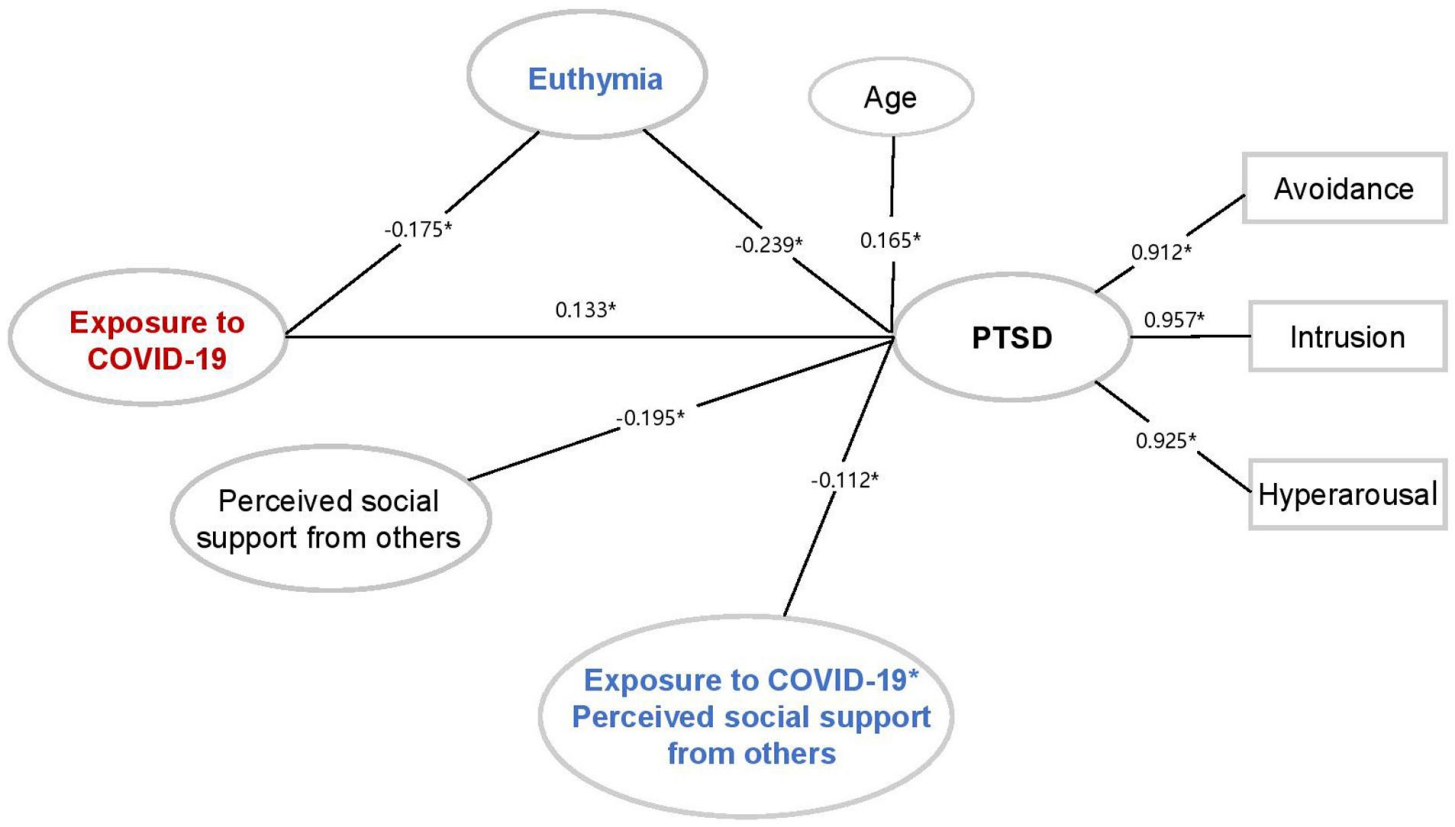
The structural equation model describing the relationship among exposure to COVID-19, euthymia, perceived social support from others, and PTSD symptoms. *p < 0.05.

To further investigate which sub-dimensions drove this moderating effect of perceived social support, we developed models based on different sources of support (i.e., family and others) and only found that perceived social support from others (*β* = −0.112, *p* = 0.029) moderated the direct effect of overexposure to PTSD symptoms, neither from family (*β* = −0.549, *p* = 0.069) (Figure 3). Besides, the model including perceived social support from others had a good fit with CFI = 0.982, TLI = 0.975, RMSEA =0.061, SRMR = 0.042. These results suggested that increasing perceived social support, especially from others, and euthymia may protect healthcare workers from falling prey to PTSD symptoms.

## Discussion

This study highlighted the high prevalence of PTSD symptoms among healthcare workers during the COVID-19 pandemic. We found that healthcare workers with higher PTSD symptoms were associated with overexposure to COVID-19 environment, worse euthymia and lower levels of perceived social support. Finally, euthymia and social support affected the effect of COVID-19 exposure on PTSD symptoms in separate mechanisms.

Our results showed that 45.37% of our participants had severe symptoms of PTSD, with the most common symptoms being intrusive thoughts and memories. Compared to previous studies, the prevalence of PTSD symptoms among healthcare workers in this study was relatively high (36), high number of infections (one of the seven provinces with more than 1,000 cases) and shortage of medical resources becoming possible influencing factors. By now, the highest reported prevalence of PTSD symptoms was as high as 71.5% in Chinese healthcare workers (5). However, the study mainly focused on healthcare workers in hospitals with fever clinics and wards for the COVID-19, and 81.3% of the participants were from Hubei province (the province with the worst outbreak in 2019). We also found that higher exposure to COVID-19 was associated with more severe PTSD symptoms. This finding was consistent with recent studies that have found that higher exposure to COVID-19 (6), like working in the clinic (37), witnessing deaths and injuries (38), treating infected patients (39) and receiving a large amount of information related to the COVID-19 pandemic (40), were associated with adverse psychological outcomes such as PTSD or depression. Overall, people who were more frequently exposed to traumatic events would be more susceptible to developing PTSD symptoms (41, 42). Notably, our study did not stop here, we further clarified the two positive factors that could alleviate PTSD symptoms influenced by overexposure to COVID-19 on healthcare workers in Shandong.

Specifically, we provided evidences supporting the buffering role of euthymia and perceived social support on releasing PTSD symptoms in healthcare workers, albeit in distinct ways. We found that the euthymia acted as a mediator in the relationship between exposure to COVID-19 and PTSD symptoms. That is, healthcare workers with higher level of exposure to the COVID-19 pandemic tended to show poorer euthymia state and more PTSD symptoms. This was similar to previous findings which observed significantly decreased psychological well-being (43), the psychological flexibility and the ability to cope with stress (44) during the COVID-19 pandemic, triggering higher levels of anxiety and depression. In other words, the threat of infection, restrictions on social activities, and close contact with infected people could pose a direct threat to individual euthymia state, which may further exacerbate trauma symptoms.

Unlike euthymia, perceived social support was not directly related to exposure to COVID-19. However, it was found to moderated the effect of overexposure to COVID-19 on PTSD symptoms. That is, when perceived social support levels were low, overexposure to COVID-19 increased the possibility of developing PTSD symptoms, but such impact was decreased when levels of perceived social support were high. These findings were in line with the conservation of resources (COR) model, which proposed that social support could broaden an individual’s resources to resist stress and protect psychological health (29). Besides, we found that only social support from others such as friends, leaders, relatives and colleagues could mitigate the effect of overexposure on PTSD symptoms. This finding provided additional evidence for the protective effect of perceived social support on psychological health (PTSD, anxiety, depression) (45, 46). Consistent with it, a study of mental health among Polish nurses also found that support from significant others was the main source of social support (47). It was likely that heavy workloads and isolation in hospitals significantly reduced their social interactions with family, which made friends, patients, colleagues and leaders became the most promising sources of social support for healthcare workers.

Despite the positive results presented in this paper, there were several limitations. Firstly, the sample size of the current study was relatively small, further studies with larger sample sizes were need to confirm our conclusions revealed in this paper. Secondly, there was heterogeneity in participants, and their relative mental states may vary depending on their different working load. For instance, previous studies have found that healthcare workers in the Intensive Care Unit (ICU) exhibited more pronounced mental health problems during the COVID-19 (48). Future research could validate the role of social support and euthymia in a more specific group of healthcare workers. Thirdly, the self-measured exposure questionnaire was designed for this study and lacked reliability and validity tests. Future studies could further optimize items and make it more reliable. Finally, our study focused on PTSD symptoms. Future studies could investigate whether the function of euthymia and social support can be generalized to other emotional distress, such as anxiety and depression.

In conclusion, this study confirmed a close correlation between overexposure to COVID-19 and PTSD symptoms, highlighting specific roles of euthymia and social support in alleviating PTSD symptoms. These results suggested that enhancing social support especially from significant others and increasing levels of euthymia in healthcare workers may be useful for the intervention of PTSD symptoms after the pandemic.

## Data availability statement

The original contributions presented in the study are included in the article/supplementary materials, further inquiries can be directed to the corresponding authors.

## Ethics statement

The studies involving human participants were reviewed and approved by the Ethics Committee of Shandong Provincial Mental Health Centre. The patients/participants provided their written informed consent to participate in this study.

## Author contributions

YuY, SH, JG, and DW designed the experiment. YuY, JG, YiY, and SH performed the study. YuY, SH, JG, DW, HW, T-FY, and YiY analyzed the results and wrote the paper together. All authors have read and approved the final version of the manuscript.

## Funding

This work was supported by the Shandong Provincial Health and Wellness Commission Pharmaceutical Science and Technology Project (202003091014). The funding agencies did not contribute to the experimental design or conclusion.

## Conflict of interest

The authors declare that the research was conducted in the absence of any commercial or financial relationships that could be construed as a potential conflict of interest.

## Publisher’s note

All claims expressed in this article are solely those of the authors and do not necessarily represent those of their affiliated organizations, or those of the publisher, the editors and the reviewers. Any product that may be evaluated in this article, or claim that may be made by its manufacturer, is not guaranteed or endorsed by the publisher.
